# Clinical Re-evaluation on Bioequivalence and Relative Bioavailability of Micronized Progesterone Hard Capsule (Yimaxin) and Micronized Progesterone Soft Capsule (Utrogestan) under Vaginal and Oral Administration Routes

**DOI:** 10.12669/pjms.37.7.3949

**Published:** 2021

**Authors:** Hanbi Wang, Meizhi Liu, Rui Chen, Chengyan Deng

**Affiliations:** 1 Hanbi Wang, Department of Obstetrics and Gynecology, Peking Union Medical College Hospital, Chinese Academy of Medical Sciences & Peking Union Medical College, National Clinical Research Center for Obstetric & Gynecologic Diseases, 1# Shuai fu yuan, Dongcheng District, Beijing 100730, China; 2 Meizhi Liu, Department of Obstetrics and Gynecology, Peking Union Medical College Hospital, Chinese Academy of Medical Sciences & Peking Union Medical College, National Clinical Research Center for Obstetric & Gynecologic Diseases, 1# Shuai fu yuan, Dongcheng District, Beijing 100730, China; 3 Rui Chen, Department of Obstetrics and Gynecology, Peking Union Medical College Hospital, Chinese Academy of Medical Sciences & Peking Union Medical College, National Clinical Research Center for Obstetric & Gynecologic Diseases, 1# Shuai fu yuan, Dongcheng District, Beijing 100730, China; 4 Chengyan Deng, Department of Obstetrics and Gynecology, Peking Union Medical College Hospital, Chinese Academy of Medical Sciences & Peking Union Medical College, National Clinical Research Center for Obstetric & Gynecologic Diseases, 1# Shuai fu yuan, Dongcheng District, Beijing 100730, China

**Keywords:** Micronized progesterone hard capsule, Micronized progesterone soft capsule, Bioequivalence, Bioavailability, Pharmacokinetics

## Abstract

**Background and Objective:**

To clinically re-evaluate relative bioavailability and bioequivalence of micronized progesterone (hard capsule) Yimaxin and micronized progesterone (soft capsule) Utrogestan under vaginal and oral administration routes.

**Methods:**

From December 2017 to June 2018, a total of 16 postmenopausal healthy women were recruited and received a total of four rounds of drug treatment with cross-over design, respectively Yimaxin and Utrogestan under vaginal and oral administration routes. Changes in the subjects’ hormone levels after medication were monitored and an endometrial biopsy after a course of treatment was performed in our hospital.

**Result:**

The Geomeans of AUC_0-t_ of Yimaxin and Utrogestan under vaginal administration route were 252.15 and 115.46, respectively, with a ratio of 2.19, and under oral administration route were 244.64 and 413.68, respectively, with a ratio of 0.59. The Geomeans of C_max_ of Yimaxin and Utrogestan under vaginal administration route were 28.11 and 12.21, respectively, with a ratio of 2.30, and under oral administration route were 53.12 and 129.85, respectively, with a ratio of 0.41.

**Conclusion:**

Yimaxin was not bioequivalent to Utrogestan. Yimaxin had higher exposure to the drug in vivo at the same dose when administered vaginally, and Utrogestan had higher exposure to the drug in vivo at the same dose when administered orally.

## INTRODUCTION

Exogenous progesterone Utrogestan and Yimaxin are two widely used natural progesterone preparations in China that play an important role in female reproduction.^1^ Especially in the fetus-protecting treatment, they can reduce the speed of electric signal transmission in uterine muscle layer and inhibit uterine contraction.^2^ The first micronized natural progesterone soft capsule (brand name: Utrogestan) was developed and marketed by French Besins since 1980.^3^ It is synthesized from a natural substance extracted from yams (Dioscoreasp).

**Fig.1:**
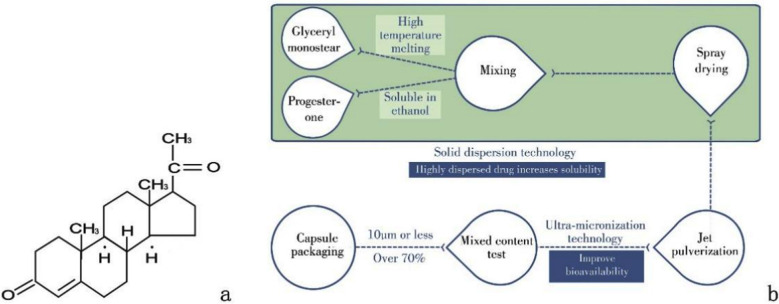
Yimaxin’s chemical structural formula (a) and process flo w diagram (b).

Yimaxin (micronized natural progesterone hard capsule) is an independent and innovative progesterone preparation from China. It was launched into Chinese market in 2004. It is a chemical synthesis of diosgenin extracted from the plant turmeric (Dioscorea nipponica). Its chemical structure is shown in Fig.1(a) and the process flow of Yimaxin is shown in Fig.1(b). After superfinely pulverizing the excipients and dispersions by supersonic airflow micronizer, the specific surface area of excipients and drugs is further increased, and the functions of the hydrotropy of excipients and the dissolution of original drugs are enhanced. Progesterone hard capsules do not contain oily excipients such as sunflower oil and soybean phospholipids, and this is not allergic to patients. Patients do not show any allergic reaction.

A meta-analysis and comparison found that both Utrogestan and Crinone were safe and effective during vaginal administration in the assisted reproductive technology.^4^ In this study, Utrogestan was used as a reference preparation to investigate the relative bioavailability of Yimaxin hard capsules to evaluate the bioequivalence of two preparations.

Despite micronized progesterone was commonly used for pregnancy-related luteal support, but the research could be interfered by the progesterone produced by corpus luteum. So we selected the postmenopausal women who would eliminate the effects of hormones secreted by functional ovaries.

## METHODS

A total of 16 Chinese healthy menopausal female volunteers aged 45-60 years were recruited in this study from December 2017 to June 2018.This study was approved by the Ethics Committee of Peking Union Medical College Hospital (HS-1451).

### Inclusion criteria

Menopausal women were recruited, with the follicle-stimulating hormone (FSH) level showing post-menopausal status, basal progesterone level less than 1.0ng/ml, body mass index of 20-25 kg/m^2^, no addiction to tobacco, alcohol or drug addiction, study subjects could communicate well with researchers, willing to cooperate in completing clinical trial, and voluntarily signed an informed consent form.

### Exclusion criteria

- Those with any abnormalities that might affect the study and any clinically significant laboratory examination abnormalities during the screening phase - Those patients who had participated in any other clinical drug trials in the last three months; had taken any steroid hormone drugs in the last three months; had allergic reactions or severe adverse reactions to the test drug; often used or were using drugs such as enzyme inducers or inhibitors that affect the drug metabolism, such as sex hormone, phenytoin, barbital, primidone, carbamazepine, rifampicin, griseofulvin; those with a medical history of thromboembolism or thrombophlebitis; serious cardiovascular problems; a medical history of any tumors; hematological system disorders; severe or frequent headaches; those who had suffered from mental disease, depression or epilepsy.

### Trial Protocol

The general information of the subjects was collected. Physical examination included vital signs, assessment of systemic organs, electrocardiogram, chest X-ray and breast ultrasound, basic sex hormone examination, gynecological examination, transvaginal ultrasound, blood, urine and blood biochemical examination. The patients were screened and enrolled by the inclusion criteria and randomized into Group-A and Group-B by the ratio of 1: 1.

Morning fasting blood was drawn to test P. The thickness of the endometrium was determined by vaginal ultrasound. The subjects orally administered Progynova 2mg, qd, from Day 1 to Day 20. On Day 11, morning fasting blood was drawn to test Estrogen (E_2_) and Progestin (P), and the thickness of the endometrium was determined by vaginal ultrasound. From Day 11 to Day 20, Group-A and Group-B additionally used Yimaxin (50mg, Zhejiang Xianju Pharmaceutical Co., Ltd. China) for vaginal medication (Group-A) or Utrogestan (100mg, Besins Manufacturing Belgium, France) for vaginal medication (Group-B), 200 mg bid (q12h) respectively. On Day 11, blood samples were collected for pharmacokinetic analysis before the first use of study drug and 0.5, 1, 1.5, 2, 3, 4, 6, 8, and 12 hours after administration. After the blood sample was collected 12 hours after the completion of administration on the same day, the study drug was administered for the second time.From Day 12 to Day 19, blood samples were collected for pharmacokinetic analysis before the first administration of each day. On Day 20, blood samples were collected for pharmacokinetic analysis before the first use of study drug and the same as the Day 11. After the blood sample was collected 12 hours after the completion of administration on the same day, the study drug was administered for the second time. On Day 21, the medication (estrogen and progesterone) was stopped, and morning fasting blood samples were collected. The endometrium was taken and sent for pathological examination (pathological specimens of the endometrium were blindly read by two pathologists from the Department of Pathology of Peking Union Medical College Hospital respectively). Fasting blood was drawn every morning for pharmacokinetic analysis until 3 days after drug withdrawal. Adverse events and concomitant medications were recorded at each visit.

The washout period was 15 days after the last administration. Adverse events and concomitant medications were recorded. After that, the subjects entered the second round of medication. In the second round of medication, Group-A and Group-B used both the progestogen drugs interchangeably vaginal route. The procedures for medication and collection of serum samples were the same as the first round.

**Fig.2:**
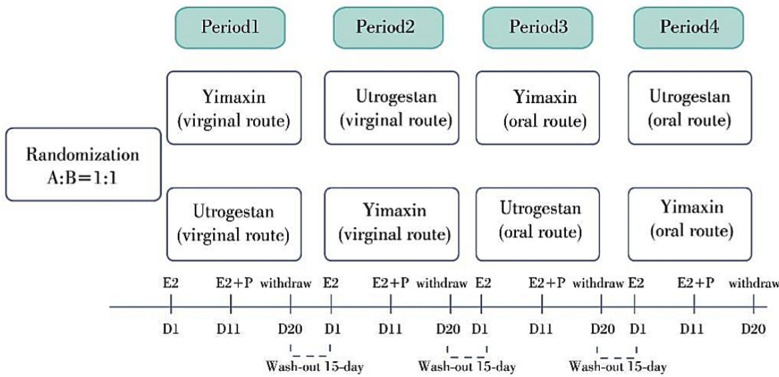
Study’s schematic diagram Note: There was a 15 days washout period between doses.

**Fig.3:**
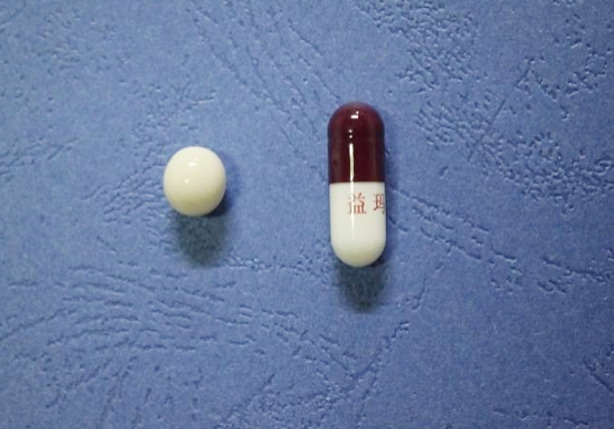
Yimaxin (right, micronized progesterone); Utrogestan (left, micronized progesterone).

In the third round of medication, Group-A and Group-B used progesterone via oral route. In the fourth round of medication, Group-A used Utrogestan orally and Group-B used Yimaxin orally. The trial procedure and the procedure for collection of serum samples were the same as the first round. Each round had a 14-day washout period. At the same time, other conditions such as adverse events and concomitant medications were collected at each follow-up. Assessment of menstrual blood volume was based on the subjective sensation of the subjects. The cross-over flow diagram was shown in Fig.2.At the end of trial, a comprehensive safety assessment was performed. The assessment content was the same as the full set of examination before enrollment. Utrogestan and Yimaxin drugs were shown in Fig.3.

### Collection and processing of blood samples and endometrium

An indwelling needle was placed on the forearm of the subjects during each intensive blood sample collection. Heparin was used to seal the tube after each blood sample was drawn, and 0.2-0.3 ml of blood was drawn and discarded before each blood sample was collected. Venous blood sample 3 ml was collected each time.

Whole blood 3 ml was collected at each time point and stored at room temperature and sample centrifugation was completed within 60 min. After mixing upside down, it was put in a centrifuge, 2000g, and centrifuged at 4°C for 10 minutes. The upper serum sample 0.5ml was taken immediately and put into a test tube, and the rest was put into a backup tube. After the end of separation operation, the serum was immediately stored upright in a refrigerator at -20±5°C for pre-freezing and temporary storage. The samples should be stored in the temporary storage refrigerator for at least two hours, and stored in the -70 °C refrigerator after the end of a sampling period. The serum concentration of the progesterone was determined by a validated electrochemical method.

After the medication (estrogen and progesterone) of each round was stopped, morning fasting blood samples were collected and the endometrial sampler (PRODIMED SAS, France) was used to collect endometrial tissues, which were immediately stored with 10% formalin and sent for pathological examination (The pathological specimens of the endometrium were read by two pathologists from the Department of Pathology of Peking Union Medical College Hospital respectively).

### Safety evaluation

The severity of all adverse events during the trial was determined based on U.S. Department of Health and Human Services, National Institutes of Health National Cancer Institute 4.03 (NCI CTCAE v4.03) (http://evs.nci.nih.gov/ftp1/CTCAE/CTCAE_4.03_2010-06-14_QuickReference_8.5x11.pdf). CTCAE classification criteria:

### Data Management and Statistical Analysis

The data were collected using a CRF form (case report form), which were entered into the database after being reviewed and verified by clinical monitors and researchers. Data collection and statistical analysis were performed by statisticians.

Systemic exposure parameters of Yimaxin (AUC_0-t_, C_max_) and Utrogestan (AUC_0-t_, C_max_) were analyzed using a mixed-effects model. Geometric mean ratios and 90% confidence intervals (90% CIs) were calculated for the systemic exposure parameters. Bioequivalence of Yimaxin in Utrogestan would be established if 90% CIs for the ratio of population geometric means of Yimaxin AUC_0-t_ and C_max_ were contained in the equivalence limits of 80%-125%. All statistical calculations were performed with WinNon lin Standard Edition Version 5.7.

The non-compartment model analysis was used to obtain the individual bioavailability parameters AUC_0-t_ and C_max_. The Mixed-effect model was performed based on the ratio of AUC_0-t_ and C_max_ for different drugs and routes of administration to obtain relative bioavailability and bioequivalence evaluations. Statistical significance was judged at a significant level of *P*=0.05.

## RESULTS

### Subject Disposition and Baseline Characteristics

A single-center randomized, open, relative bioavailability and bioequivalence study of vaginal and oral Yimaxin compared with Utrogestan was conducted. The demographic information of 16 subjects was shown in Table-I. Blood pressure, heart rate, and body temperature were not significantly different before and after the study.

**Table I:** Summary of demographic characteristics of study subjects.

	*Mean ± SD*	*Min*	*Max*
Age (years)	53.4 ± 3.7	48	59
Menopausal period (years)	4.7 ± 2.5	1	10
Height (cm)	160.47 ± 4.49	150	172
Weight (kg)	58.59 ± 4.84	48	68
BMI (kg/m^2^)	36.49 ± 2.55	31.65	41.21

### Relative Bioavailability and Bioequivalence

The AUC_0-t_ and C_max_ data of 16 subjects after vaginal and oral administration of Yimaxin and Utrogestan were shown in Table-II and Table-III.

**Table II:** Bioequivalence Evaluation of Yimaxin for comparison with Utrogestan after vaginal administration.

	*Yimaxin*	*Utrogestan*
Parameter Statistics	n=16	n=16
AUC_0-t,_ng·h/ml		
Geometric mean	252.15	115.46
Geometric mean ratio	2.19	-
90% CI	(1.92-2.49)	-
C_max,_ng/ml		
Geometric mean	28.11	12.21
Geometric mean ratio	2.30	-
90% CI	(2.06-2.57)	-

**Table III:** Bioequivalence Evaluation of Yimaxin for comparison with Utrogestan after oral administration.

	*Yimaxin*	*Utrogestan*
Parameter Statistics	n=16	n=16
AUC0-t_ ,_ng·h/ml		
Geometric mean	244.64	413.68
Geometric mean ratio	0.59	-
90% CI	(0.46-0.76)	-
C_max,_ng/ml		
Geometric mean	53.12	129.85
Geometric mean ratio	0.41	-
90% CI	(0.27-0.62)	-

The Geometric means of the AUC_0-t_ of Yimaxin and Utrogestan after vaginal administration were 252.15 ng•h/ml and 115.46 ng•h/ml, respectively as seen in Table-II, and the Geometric mean ratio of the two was 2.19 (90% CI 1.92-2.49). The Geometric means of C_max_ were 28.11 ng/ml and 12.21 ng/ml, respectively, and the Geometric mean ratio of the two was 2.30 (90% CI 2.06- 2.57).

The Geometric means of the AUC_0-t_ of Yimaxin and Utrogestan after oral administration were 244.64 ng•h/ml and 413.68 ng•h/ml, respectively, and the Geometric mean ratio of the two was 0.59 (90% CI 0.46- 0.76). as shown in Table-III. The Geometric means of C_max_ were 53.12 ng/ml and 129.85 ng/ml, respectively, and the Geometric mean ratio of the two was 0.41 (90% CI 0.27-0.62).

### Safety

There were no adverse events in vaginal administration of Yimaxin and Utrogestan. However, when both drugs were administered orally, complaints of various degrees of discomfort were reported, as shown in Table-IV.

**Table IV:** Number and percentage of subjects with TEAEs by system and organ class.

	Treatment after oral administration	

	*Yimaxin (n=16), n (%)*	*Utrogestan (n=16), n (%)*
Any AEs	12(75.0)	11(68.8)
AEs by severity		
Mild	6 (37.5)	8 (50.0)
Moderate	6 (37.5)	3 (18.8)
Severe	0	0
AEs reported by subjects		
Dizziness	10 (62.5)	8 (50.0)
Somnolence	7 (43.8)	6 (37.5)
AEs by duration (minutes)		
15	1 (6.3)	-
30	5 (31.3)	6 (37.5)
60	3 (18.8)	5 (31.3)
90	2 (12.5)	-
120	1 (6.3)	-

TEAE: treatment-emergent adverse event.

## DISCUSSION

There are many studies on Utrogestan, including those with micronized progesterone as a reference drug.^5^-^8^ A comparative experiment between Crinone and Utrogestan found that Crinone was better tolerated than Utrogestan, but there were no significant differences in efficiency and safety.^9^ The comparison between dydrogesterone and micronized progesterone showed that both could reduce vascular resistance in patients with recurrent abortion and were effective in improving uterine blood flow.^10^ Compared with intramuscular progesterone and oral relative bioavailability was about 10% of intramuscular progesterone.^11^ Based on the above-mentioned full study on Utrogestan and affirmation of clinical efficacy and safety, Utrogestan was selected as a reference drug for comparative study of Yimaxin in this study.

The parameters commonly used for evaluation are AUC_0-t_ and C_max._ Bioequivalence usually uses a single-dose cross-administration of test drug and control drug.^12^It can be seen from results that the ratio of AUC_0-t_ and C_max_ of Yimaxin and Utrogestan, whether in the vaginal route or the oral route, and the 90% CIs of the geometric mean ratios were out of the bioequivalence limit of 80% - 125% for comparison. Therefore, the two drugs, neither vaginal route nor oral route are bioequivalent, that is, the two drugs are not similar.

This study used the self-crossover before-and-after control method in order to minimize the effect of individual differences as much as possible, and ensure the scientificity and reliability of experimental data. In addition, the diet on the day of continuous blood collection was uniformly supplied, to a certain extent, to avoid the effect of food on the results from the perspective of diet. This study calculated the relative bioavailability. Results in the study suggested that the two were not bioequivalent. Yimaxin’s pharmaceutical manufacturer might consider improving the process technology of the drug carrier.

The endometrial pathological test was performed after drug discontinuation. Endometrial thickness before and after medication of the four cross-rotation experiments was shown in Table-V. Endometrial pathological biopsy with vaginal administration of Yimaxin showed that 15 patients saw endometrium during secretory period (endometrial pathology was in 10- 11 days of the luteal phase) except one for too little endometrium. After vaginal administration of Utrogestan, 10 subjects had excessive menstrual blood volume, one case could not be evaluated for little tissue, one case showed atrophy of glands, slightly large interstitial cells, and the remaining 14 cases showed secretory period. After oral administration of Yimaxin, four subjects had excessive menstrual blood volume, seven subjects had moderate, and five subjects had little. Endometrial pathological examination showed that 14 cases showed secretory period endometrium except two for too few endometrium. After oral administration of Utrogestan, seven subjects had excessive menstrual blood volume, six cases had moderate, and three cases had little. Endometrial pathological examination showed that 14 subjects showed secretory period endometrium except two for little endometrium. The final pathological results reflected the pathological characteristics of the endometrium during the secretion period, indicating that whether Yimaxin or Utrogestan, the route of action of the two drugs did not affect the effect on the endometrium and both could play a good role in endometrial conversion.

**Table V:** Endometrial thickness of 16 subjects before and after medication of the four crossover experiments (mean±SD (mm)).

	Vaginal Yimaxin		Vaginal Utrogestan		Oral Yimaxin		Oral Utrogestan	

	*Before administration*	*After administration*	*Before administration*	*After administration*	*Before administration*	*After administration*	*Before administration*	*After administration*
Endometrial thickness	3.01±0.94	7.34±1.96	2.21±0.82	9.78±1.77	3.58±1.10	8.59±2.66	2.54±0.85	7.33±1.84

This study found that all subjects had no significant adverse reactions through the vaginal route on the contrary in the oral route, which were mainly manifested as headache, dizziness, drowsiness, and even affected normal daily activities in severe cases. The side effects of this experiment were consistent with the performance in other reported drug studies.^13^

### Limitations of the study

The limitation of this study include the use of micronized progesterone in menopausal women, in order to avoid the interference of progesterone secreted by the corpus luteum, we had selected the postmenopausal women instead of reproductive age women. But the micronized progesterone was commonly used in reproductive age women, there should be some differences between the two groups.

## CONCLUSION

In summary, from the study and observation of Yimaxin and Utrogestan, it was found that the bioavailability of Yimaxin and Utrogestan was not equivalent. Yimaxin had higher exposure to the drug in vivo at the same dose when administered vaginally, and Utrogestan had higher exposure to the drug in vivo at the same dose when administered orally. Although the two drugs were administered vaginally without any side effect, the oral route showed significantly different, such as vertigo and somnolence. When patients using micronized natural progesterone have obvious side effect, choosing the vaginal route of administration instead of the oral route of administration might be an alternative method.

### Authors’ contributions:

**HW, CD:** Made substantial contributions to the conception and design of the study and wrote the original draft of the manuscript.

**ML, RC:** Were responsible for data acquisition.

All authors read and approved the final version of the manuscript and are accountable for integrity of research..
